# Urinary Collectrin (TMEM27) as Novel Marker for Acute Kidney Injury

**DOI:** 10.3390/life12091391

**Published:** 2022-09-06

**Authors:** Sahra Pajenda, Ludwig Wagner, Daniela Gerges, Harald Herkner, Tamar Tevdoradze, Karl Mechtler, Alice Schmidt, Wolfgang Winnicki

**Affiliations:** 1 Department of Internal Medicine III, Division of Nephrology and Dialysis, Medical University of Vienna, 1090 Vienna, Austria; 2 Department of Emergency Medicine, Medical University of Vienna, 1090 Vienna, Austria; 3 Department of Renal Replacement Therapy, Nephrology and Transplantation, Tbilisi State Medical University and Ingorokva High Medical Technology University Clinic, Tbilisi 0144, Georgia; 4 ProtChem Facility, IMP-IMBA, Research Institute of Molecular Pathology, 1030 Vienna, Austria

**Keywords:** collectrin, biomarker, acute kidney injury

## Abstract

Acute kidney injury (AKI) is a leading complication in hospitalized patients of different disciplines due to various aetiologies and is associated with the risk of chronic kidney disease, the need for dialysis and death. Since nephrons are not supplied with pain signals, kidney injury is mostly diagnosed by serum creatinine with a time delay. Recent work has shown that certain urinary biomarkers are available for early detection of AKI. In total, 155 subjects, including 102 patients with AKI at various stages and 53 subjects without AKI, were enrolled, and their course and laboratory data were recorded. Urinary collectrin (TMEM27) was measured by a commercially available ELISA assay. Changes in serum creatinine were used to determine AKI stage. Patients with AKI presented with significantly lower levels of urinary collectrin compared to patients without AKI (1597 ± 1827 pg/mL vs. 2855 ± 2073; *p* = 0.001). Collectrin was found to inversely correlate with serum creatinine and stages of AKI. Collectrin levels were lowest in AKI stage III (1576 ± 1686 pg/mL; *p* = 0.001) and also significantly lower in stage II (1616 ± 2148 pg/mL; *p* = 0.021) and stage I (1630 ± 1956 pg/mL; *p* = 0.019) compared to subjects without AKI. An optimal minimum collectrin cut-off value of 1606 [95% CI 1258 to 1954] pg/mL was determined to detect AKI. In conclusion, urinary collectrin represents an indicator of AKI that, unlike all other established AKI biomarkers, decreases with stage of AKI and thus may be associated with a novel pathogenic pathway.

## 1. Introduction

Acute kidney injury (AKI) is an abrupt onset of deterioration in kidney function, associated with significant morbidity and mortality. With increased stage of AKI, the risk of death and need for renal replacement therapy (RRT) increases. Even after apparent recovery of AKI, there is a significant risk of chronic kidney disease (CKD), cardiovascular disease and death. This highlights the importance of research on test methods such as new biomarkers for the early diagnosis of AKI. Established clinically used markers such as serum creatinine (sCr) and blood urea nitrogen (BUN) often show an AKI with considerable delay after the causative noxious event. Better clinical outcomes may be achieved by detecting AKI early and taking appropriate action [1,2] before harmful processes disturb energy reservoirs and cellular processes at specific sites of the nephron [3]. This early diagnosis would be particularly important for kidneys as they lack clinical signs such as pain in the phase of cell injury. Several potential AKI-specific marker proteins have been identified in the past which were thought to increase specifically in urine following toxic or noxious insults at the nephron. In addition to KIM1 [4], neprilysin [5], NGAL [6], cystatin [7] and IL18 [8], the NephroCheck parameters TIMP2 and IGFBP7 [9,10] can be measured in a bedside test. The latter are expected to be the fastest diagnostic markers available to detect AKI. In contrast, serum creatinine reflects kidney injury with a delay of up to 24 h.

Novel methods to measure the human urinary proteome have opened new paths to discover further peptides or proteins indicative of kidney injury [11,12,13,14].

Collectrin (also named TMEM27) represents a type I membrane protein which shares the C-terminal domain with the angiotensin-converting enzyme-2 [15]. There is already extensive knowledge about the physiological functions of collectrin [16,17,18,19,20]. In vitro and in vivo studies have shown that collectrin plays an important role in amino acid transport in the kidney through heterodimerizing with B^0^AT1 [16,18]. Furthermore, collectrin contributes to the formation of primary cilia [21] and has been discussed to be involved in the pathogenesis of SARS-CoV-2. Collectrin has been suggested to create binding sites for SARS-CoV-2 through its heterodimerizing function, thereby affecting primary cilia, contributing to AKI pathogenesis [22].

The aim of this study was to investigate whether urinary excretion of collectrin is altered in patients with AKI. Thus, we focused on patients admitted to our Division of Nephrology to determine levels of urinary collectrin and to evaluate its association with the incidence and grade of AKI, as well as to test its diagnostic and prognostic value.

## 2. Methods and Patients

### 2.1. Study Design

This retrospective study was conducted to evaluate the collectrin excretion in urine among patients with AKI at the Division of Nephrology and Dialysis at the Medical University of Vienna. Urinary collectrin levels were compared in patients with AKI (stages I, II and III), patients without AKI with CKD and in healthy controls. Acute kidney injury was assessed by changes in serum creatinine as defined by KDIGO guidelines [23]. All urine samples were centrifuged at 3000 rpm for 10 min to pellet cells and debris and stored at −80 °C in aliquots until analysis. Samples were blinded by numbers for anonymization.

### 2.2. Study Population

A total of 155 subjects were included in this study. These consisted of 102 patients (female/male = 42/60, mean age 57.6 ± 17.4 years) treated for AKI at our center, and 53 control subjects (female/male = 28/25, mean age 54.3 ± 15.4 years) without AKI. AKI and CKD patients were consecutively recruited, and healthy volunteers were included at the start of parameter analysis (Supplementary Figure S1). Blood and urine samples of patients were collected at the time of admission and during the inpatient stay. The primary sample collection was initiated in June 2011. All study patients were tested at the date of admission for collectrin excretion in urine and monitored for serum creatinine, BUN and estimated glomerular filtration rate (eGFR) until discharge. These also included intensive care unit (ICU) patients who had a urine collection from the upper chamber of the urine collection device every morning that was no older than 2 h. None of the included patients had experienced SARS-CoV-2 infection.

### 2.3. ELISA Testing

The ELISA kit (Human Collectrin, CSB-EL0233823HU) from CUSABIO Biotech Co. Wuhan, China was performed as indicated in the test manual. In short, human urine was thawed and brought to room temperature. A total of 50 µL of diluted urine (1:3 or 1:10 with PBS) was applied to the test well which was followed by adding 50 µL of conjugate. After brief rotation on a rotating platform, the plate was incubated at 37 °C for 60 min. Following a washing step with the provided 1x wash buffer, the two-component substrate (TMB) was loaded onto the plate and allowed to react for 15 min at 37 °C. The reaction was stopped by adding 50 µL of stop solution, and it was read at 450 nm using an ELISA reader; concentrations were computed using the standard curve provided.

### 2.4. Data Processing and Statistical Analyses

We presented categorized data as absolute counts with relative frequencies. Continuous data were reported as means ± standard deviation (SD). The Mann–Whitney U test, median test or Fisher’s exact test was utilized to perform comparisons of baseline variables among study groups. The one-sample Kolmgorov–Smirnov Test was used to formally test for normality.

In this study, we calculated standard metrics for diagnostic test accuracy, including sensitivity and specificity of collectrin values (index test) to independently detect patients with AKI (reference standard). We assessed the optimal cut-off value by maximizing the sensitivity/specificity product by the Liu’s method [24], employing the adjustment approach proposed by Fluss et al. [25]. Furthermore, we estimated the non-parametric c-statistic for collectrin on AKI based on bootstrapped standard errors using 1000 replications for the calculation of 95% confidence intervals. In addition, we assessed the association between collectrin levels and creatinine. In order to make use of repeated measurements, we used random effects linear regression including the patient identifier as the panel identifier and creatinine as the predictor variable for collectrin.

For prognostic assessment of collectrin (natural logarithm) for end-stage renal disease (ESRD) or death, multivariable logistic regression analyses were employed. Further co-variables were gender, age (years), baseline eGFR (mL/min), peak CRP (mg/dL), peak white blood count (G/L), history of kidney transplantation and need for ICU admission. We reported the estimates from the regression analysis with a 95% confidence interval and the corresponding *p*-values from the Wald test.

Data management and analysis were performed by Microsoft Excel, Stata 17 and GraphPad Prism 8. All tests performed were two-sided, and a *p*-value of less than 0.05 was considered significant.

## 3. Results

### 3.1. Study Population

In this study, the demographic data, laboratory parameters and biomarkers for AKI measured by ELISA assay and chemical methods were examined in 155 individuals. These included 27 patients with AKI stage I, 18 subjects with AKI stage II, 57 patients with AKI stage III and 53 individuals without AKI. The latter included 32 patients with CKD and 21 healthy volunteers as controls. Demographic and clinical data of the entire study cohort are shown in Table 1.

### 3.2. Urinary Collectrin Values Depending on Kidney Function

Urine collectrin ELISA analyses were performed on samples from 155 subjects, including 102 patients with AKI at various stages and 53 subjects without AKI, comprising 32 patients with stable CKD and 21 subjects without renal pathology. Collectrin was found to correlate inversely with serum creatinine and stages of AKI.

**Figure 1:**
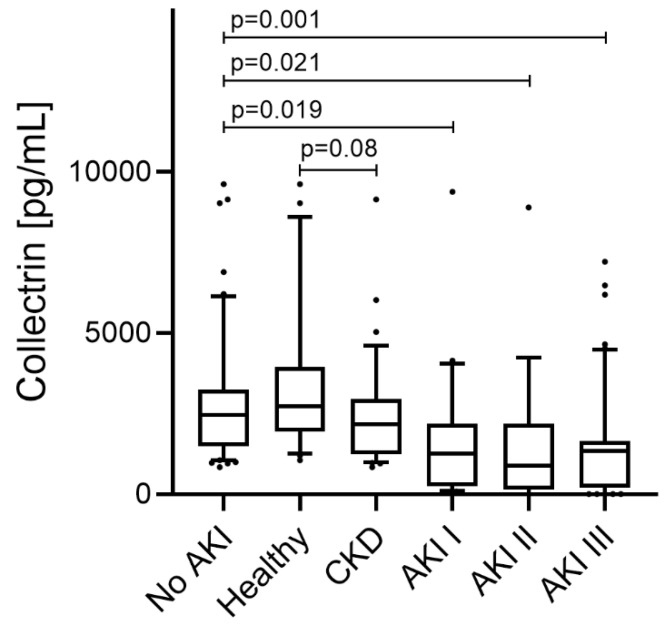
Urinary levels of collectrin in subjects with and without AKI. Minimum urinary collectrin values and association with stage of AKI. Minimum collectrin levels of patients with AKI stage I (1630 ± 1956 pg/mL; *p* = 0.019), AKI stage II (1616 ± 2148 pg/mL; *p* = 0.021) and AKI stage III (1576 ± 1686 pg/mL; *p* = 0.001) were significantly lower when compared with subjects without AKI (2855 ± 2073 pg/mL). Among patients without AKI, there was no difference between collectrin levels of healthy volunteers and patients with chronic kidney disease (CKD); 3468 ± 2452 pg/mL vs. 2453 ± 1706 pg/mL, *p* = 0.08.

Overall, patients with AKI presented with significantly lower levels of urinary collectrin compared to patients without AKI (1597 ± 1827 pg/mL vs. 2855 ± 2073; *p* = 0.001). Urinary collectrin levels, depending on renal function, respectively, and the AKI stage are listed in Table 2 and shown in Figure 1.

Furthermore, there was no association between urinary collectrin values and AKI aetiology (infection-related: 1617 ± 1940 pg/mL, hypovolemic state: 1447 ± 2083, postrenal: 2616 ± 2631, intrinsic: 1417 ± 1332, undefined: 2316 ± 895; *p* = 0.24).

Urine collectrin levels were log normally distributed. Multiple regression analysis revealed no association between minimum collectrin and gender, baseline eGFR, peak serum C-reactive protein, peak white blood count and need for ICU admission, yet there was an association with age (Table 3). 

For the prognostic assessment of urinary collectrin levels, a multivariate analysis adjusting for age, gender, baseline eGFR, peak CRP, white blood cell count, history of kidney transplantation and need for ICU admission was performed. Thereby, with each level increase of ln_collectrin, the risk of ESRD or death decreased by 29% (OR 0.713 [95 CI 0.519–0.981]; *p* = 0.04).

Collectrin and creatinine were inversely correlated, with a high intra-patient correlation. With each increase of creatinine by 1 unit mg/dl, collectrin decreased by 131 pg/mL ([95 CI −256 to −5]; *p* = 0.04).

### 3.3. Sensitivity and Specificity of Urinary Collectrin for Diagnosis of AKI

Receiver operating characteristic (ROC) analysis was performed to evaluate whether urinary collectrin was a valuabale marker for the diagnosis of acute kidney injury.

**Figure 2:**
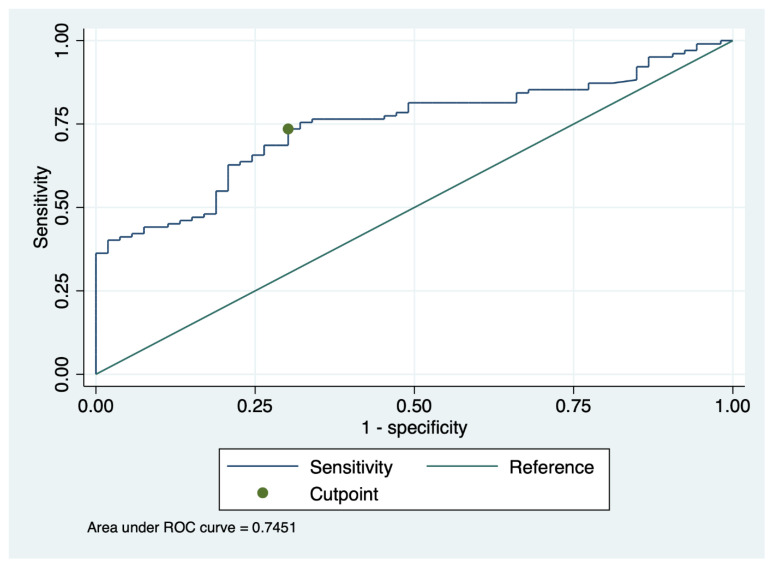
Receiver operating characteristic (ROC) analysis of urinary collectrin in subjects without and with AKI. The cut-off level of 1606 pg/mL of minimum collectrin had a sensitivity of 0.74 and a specificity of 0.70 for detecting AKI.

The optimal minimum collectrin cut-off level to distinguish between AKI and non-AKI is 1606 [95% CI 1258 to 1954] pg/mL, with a sensitivity and specificity of the assay of 0.74 and 0.70 and an AUC of 0.75 [95% CI 0.67 to 0.82; *p* < 0.01] (Figure 2).

Our findings indicate that a minimum collectrin (trough) level can be used to differentiate AKI from non-AKI.

## 4. Discussion

In this study, the excretion of soluble collectrin in human urine was investigated in patients with AKI compared to individuals without AKI. Strikingly, this parameter is decreased in AKI. This makes collectrin the first parameter identified to date that is decreased in urine in AKI, for a reason not yet known. This is in contrast to all other established AKI-specific biomarkers such as the NephroCheck parameters TIMP2 and IGFBP7 [9,10], neprilysin [5], NGAL [6], cystatin [7], KIM1 [4] and IL18 [8], which increase during AKI due to cell lesion or cell membrane injury. These protein AKI biomarkers, with the exception of KIM-1, do have the drawback that some may pass from the blood into the urine in AKI due to impairment of the glomerular membrane barrier and thus do not specifically originate from the kidney. In contrast, the dynamics of collectrin, which decreases in AKI, is not affected by a dysfunctional slit membrane. However, combining the above biomarkers with collectrin in a novel device could increase the diagnostic potential and the ability to predict progression and outcomes.

Since collectrin is highly expressed at the proximal tubular brush border and apical membrane [16,18,21], the most common site of AKI etiopathology, the protein is expected to increase in urine when renal injury occurs. Cellular proteins can usually leak into the tubular lumen and be excreted in the urine due to cell membrane injury. The opposite dynamics of collectrin demonstrated in this study need further explanation.

Most likely, this effect is caused by a lack of collectrin trafficking into the primary cilia and changes in trafficking of vesicles into which collectrin has been shown to be involved by protein–protein interaction [21]. Protein trafficking is energy-dependent, and previous authors have suggested that this may be disrupted when energy stores in the tubular epithelia are depleted, particularly in the proximal tubule, where the highest energy expenditure [26] is required for physiological function. This could explain the inverse dynamics of collectrin compared with other AKI biomarkers, which is unique in its course during AKI. In addition, collectrin is a single transmembrane domain protein involved in amino acid transport and therefore should not appear in fluid phase. There might be two exceptions: first, when still included in the well-known exosomes originating from the brush border; but this has not been identified so far. Secondly, the protein comprises a hydrophilic extracellular domain which is known to be shaded from pancreatic beta cells [27].

In our study, we could clearly demonstrate that the collectrin peptide is contained in urine. Therefore, the most likely underlying process is that the protein is cleaved from the membrane by a specific protease, and the resultant cleavage product has a yet unknown role in urine. The question still remains which proteases are involved in the cleavage process and why this process is inhibited when an injurious insult occurs at the proximal tubule or collecting duct.

Testing for urinary collectrin may have further implications for experimental animal models. In this line, pharmacotherapeutic compounds in the test phase and surgical interventions and factors influencing haemodynamics could be evaluated for their effect in causing subclinical or clinical AKI. Furthermore, transgenic models that overexpress or have knocked out proteins could be studied for the impact on susceptibility to AKI [28]. Overall, these considerations provide further, albeit partially hypothetical, insight into the complex and multifaceted pathogenesis of AKI.

The main limitation of this study is its retrospective design. There is also a heterogeneous control group of patients with CKD and healthy individuals. On the other hand, the study is strengthened by a large sample size of a well-characterized cohort of patients with AKI of different stages, allowing clear results.

## 5. Conclusions

Urinary collectrin is a novel biomarker for acute kidney injury that, in contrast to all other known AKI biomarkers, decreases during AKI. Collectrin could therefore be associated with a new pathogenic pathway. Beyond further pathomechanistic studies, prospective studies are needed to test its diagnostic and prognostic value and to determine whether specific types of AKI can be identified, particularly by collectrin.

## Figures and Tables

**Table 1:** Demographic and clinical data of the study population.

Characteristic	Subjects without AKI (n = 53)	Subjects with AKI (n = 102)	*p*-Value
**Age (years)**	54.3 ± 15.4	57.6 ± 17.4	0.25
**Gender** **–number (%)**			
-Male	25 (47)	60 (59)	0.18
-Female	28 (53)	42 (41)	
**Unit of admission** **–number (%)**			
-Intensive care unit	14 (26)	19 (19)	0.30
**Coexisting or prior illness–number (%)**			
-Arterial hypertension	32 (60)	71 (70)	
-Diabetes mellitus	10 (19)	30 (29)	0.21
-Cardiovascular disease	20 (38)	45 (44)	0.18
-Congestive heart failure	9 (17)	15 (15)	0.49
-Glomerulonephritis	3 (6)	10 (10)	0.82
**Acute Kidney Injury [23]–number (%)**			0.54
-No AKI	53 (100)		
-AKI stage I		27 (26)	
-AKI stage II		18 (18)	
-AKI stage III		57 (56)	
**AKI aetiology** **–number (%)**			
-Infection-related		44 (43)	
-Hypovolemic state		19 (19)	
-Postrenal/obstructive		6 (6)	
-Intrinsic		31 (30)	
-Undefined		2 (2)	

Plus/minus readings indicate mean values ± standard deviation. The numbers in brackets represent the percentage. Acronyms: AKI, acute kidney injury; eGFR, estimated glomerular filtration rate; KDIGO, Kidney Disease: Improving Global Outcomes.

**Table 2:** Clinical patient characteristics and levels of urinary collectrin as AKI biomarker.

	Healthy Subjects	CKD	AKI I	AKI II	AKI III	*p*-Value
Number of study participants	21	32	27	18	57	
**Characteristics**						
-Age (years)	46.1 ± 15.5	59.7 ± 13.0	56.9 ± 20.9	57.7 ± 16.1	58.0 ± 16.2	0.45
-Gender—male number (%)	9 (43)	16 (50)	14 (52)	8 (44)	38 (67)	0.14
**Laboratory parameters**						
-Urine protein/creatinine ratio (mg/g)	190 ± 9	1680 ± 2273	1356 ± 1447	953 ± 841	1718 ± 3446	0.21
-Urine albumin/creatinine ratio (mg/g)	20 ± 10	1159 ± 1701	668 ± 809	475 ± 556	868 ± 1722	0.99
-HbA1c	5.62 ± 0.16	5.86 ± 1.03	5.80 ± 1.13	5.87 ± 0.45	5.79 ± 0.93	0.73
**Baseline renal function parameters**						
-Baseline serum creatinine (mg/dL)	0.64 ± 0.15	2.35 ± 1.35	1.47 ± 0.86	1.36 ± 0.64	1.39 ± 0.80	0.52
-Baseline eGFR (mL/min)	113.21 ± 18.87	37.94 ± 24.88	56.27 ± 32.91	60.23 ± 33.47	62.88 ± 29.98	0.46
**Biomarker for acute kidney injury**						
-Minimum urine collectrin levels (pg/mL)	3468 ± 2452	2453 ± 1706	1630 ± 1956	1616 ± 2148	1576 ± 1686	0.001

Plus/minus readings indicate mean values ± standard deviation. The numbers in brackets represent the percentage. Acronyms: AKI, acute kidney injury; CKD, chronic kidney disease; eGFR, estimated glomerular filtration rate.

**Table 3:** Association between clinical variables and urinary collectrin values in patients with AKI.

Variable	Regression Coefficient (95% CI) with Minimum Collectrin Adjusted ^a^	*p*-Value
Gender (male)	0.143 (−0.421 to 0.707)	0.62
Age (years)	−0.018 (−0.035 to −0.002)	0.03
Baseline eGFR (mL/min)	−0.004 (−0.014 to 0.006)	0.41
Peak C-reactive protein (mg/dL)	0.005 (−0.024 to 0.033)	0.74
Peak white blood count (G/L)	0.016 (−0.022 to 0.055)	0.41
Need for ICU admission	−0.760 (−1.534 to 0.015)	0.06
History of renal transplantation	−0.468 (−1.084 to 0.148)	0.14

^a^ multivariable adjustment for gender (male/female), age (years), baseline eGFR (mL/min), peak serum C-reactive protein, peak white blood count, need for ICU admission and history of renal transplantation. CI, confidence interval; eGFR, estimated glomerular filtration rate; ICU, intensive care unit.

## Data Availability

The authors confirm that the data supporting the findings of this study are available within the article and/or its Supplementary Materials.

## Supplementary Materials

The following supporting information can be downloaded at: https://www.mdpi.com/article/10.3390/life12091391/s1, Figure S1: Flowchart with inclusion and exclusion criteria.
