# Supplementary material for: Urinary Collectrin (TMEM27) as Novel Marker for Acute Kidney Injury
Source: Life (Basel). 2022 Sep 6;12(9):1391. doi: 10.3390/life12091391 (PMC9503639; doi:10.3390/life12091391)
Supplement: Supplementary file 1 [file life-12-01391-s001.zip › life-1900772-supplementary.pdf]

life

*Supplementary Materials*

# Urinary Collectrin (TMEM27) as Novel Marker for Acute Kidney Injury

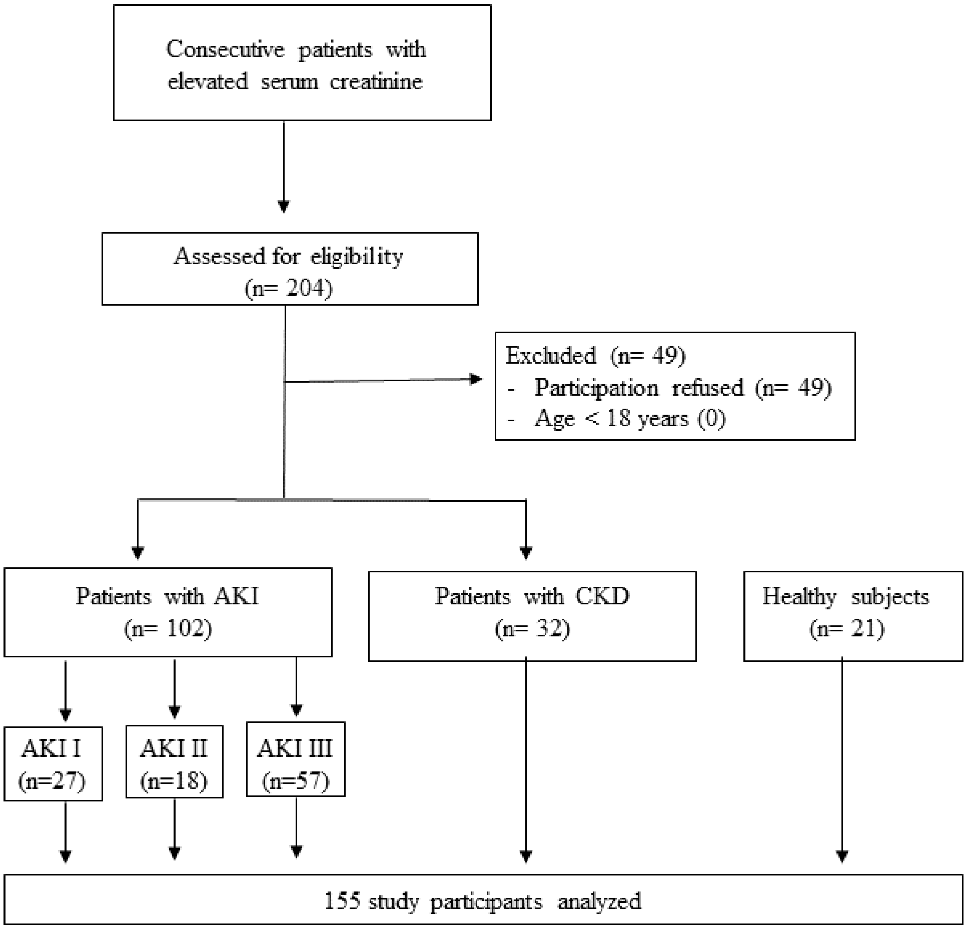


**Figure S1.** Flowchart with inclusion and exclusion criteria.
